# Using publicly available transcriptomic data to identify mechanistic and diagnostic biomarkers in azoospermia and overall male infertility

**DOI:** 10.1038/s41598-022-06476-1

**Published:** 2022-02-16

**Authors:** Temidayo S. Omolaoye, Mahmood Yaseen Hachim, Stefan S. du Plessis

**Affiliations:** 1 Department of Basic Sciences, College of Medicine, Mohammed Bin Rashid University of Medicine and Health Sciences, Dubai, UAE; 2 Division of Medical Physiology, Faculty of Medicine and Health Sciences, Stellenbosch University, Tygerberg, South Africa

**Keywords:** Diagnostic markers, Reproductive disorders

## Abstract

Azoospermia, which is the absence of spermatozoa in an ejaculate occurring due to defects in sperm production, or the obstruction of the reproductive tract, affects about 1% of all men and is prevalent in up to 10–15% of infertile males. Conventional semen analysis remains the gold standard for diagnosing and treating male infertility; however, advances in molecular biology and bioinformatics now highlight the insufficiency thereof. Hence, the need to widen the scope of investigating the aetiology of male infertility stands pertinent. The current study aimed to identify common differentially expressed genes (DEGs) that might serve as potential biomarkers for non-obstructive azoospermia (NOA) and overall male infertility. DEGs across different datasets of transcriptomic profiling of testis from human patients with different causes of infertility/ impaired spermatogenesis and/or azoospermia were explored using the gene expression omnibus (GEO) database. Following the search using the GEOquery, 30 datasets were available, with 5 meeting the inclusion criteria. The DEGs for datasets were identified using limma R packages through the GEO2R tool. The annotated genes of the probes in each dataset were intersected with DEGs from all other datasets. Enriched Ontology Clustering for the identified genes was performed using Metascape to explore the possible connection or interaction between the genes. Twenty-five DEGs were shared between most of the datasets, which might indicate their role in the pathogenesis of male infertility. Of the 25 DEGs, eight genes (THEG, SPATA20, ROPN1L, GSTF1, TSSK1B, CABS1, ADAD1, RIMBP3) are either involved in the overall spermatogenic processes or at specific phases of spermatogenesis. We hypothesize that alteration in the expression of these genes leads to impaired spermatogenesis and, ultimately, male infertility. Thus, these genes can be used as potential biomarkers for the early detection of NOA.

## Introduction

Male infertility, which can simply be defined as the inability of a male to impregnate a fertile female after 12 months or more of regular sexual intercourse^1^, accounts for 50% of the total cases of infertility^2^. Using the World Health Organization diagnostic criteria for male infertility^3^, studies have elucidated the following as some of the attributable risk factors of male infertility: azoospermia, oligozoospermia, asthenozoospermia, teratozoospermia, or combinations thereof, amongst others^4–6^. The impact of systemic diseases, endocrine abnormalities, congenital abnormalities, acquired testicular damage, varicocele, formation of anti-sperm antibodies, male accessory gland infection have also been implicated to play a part in deteriorating male fertility^7^. The conventional semen analysis merely provides information on the male’s fertility potential and does not provide information on the etiology. In order to explore the underlying cause, the medical history, physical examination and e.g., hormonal analysis should also be taken into consideration. Despite conducting these investigations, the cause of male infertility remains unanswered in more than 25% of cases are classified as idiopathic or of unknown aetiology^8^.

Azoospermia, which can be defined as the absence of sperm in the semen after analysing two consecutive samples is prevalent in about 10–15% of infertile men^9^. Azoospermia can be categorized into two types: (i) obstructive azoospermia (OA), accounts for 40% of azoospermic cases, and is caused by the blockage or missing connection in the epididymis, vas deferens, or anywhere along the reproductive tract and usually, normal spermatogenesis may occur, while, (ii) nonobstructive azoospermia (NOA) which accounts for 60% of azoospermia cases occurs due to impaired spermatogenesis, genetic deletions or testicular dysfunction^10^. The aetiology of azoospermia can be pre-testicular (endocrine disorders), testicular (Sertoli-cell only syndrome (SCOS), testicular torsion, varicocele, orchitis, toxins), and/or post-testicular (ejaculatory disorders). The latter is primarily seen in OA and can be treated by surgically removing or repairing the blockage. However, since NOA is mainly caused by defective or impaired spermatogenesis and about 25% of NOA cases remain classified as idiopathic^11^, it is pertinent to identify the essential genes involved in this process as potential biomarkers.

Spermatocytogenesis, spermiogenesis and spermiation are developmental processes regulated by an enormous network of endocrine, paracrine, and genetic communications. The functional interdependence of these delicate events is essential for overall male fertility. Findings have elucidated that the functional interplay between the germ cells and the Sertoli cells also plays a vital role in regulating spermatogenesis^12,13^. The disturbance of these processes (spermatocytogenesis, spermiogenesis and spermiation, altogether known as spermatogenesis), may result in various aberrations, ranging from defects in germline differentiation, abnormal spermatid formation to azoospermia (NOA specifically)^14^.

To better understand the aetiology of male infertility, studies are now investigating the genetic structure of each process of spermatogenesis and how alterations in the genetic composition can cause male infertility^15–18^. For instance, Xia et al. reported the presence of differentially regulated genes important for spermatogenesis in the testes of rats. The proteins include cell junction-associated proteins, transcription factors pertinent to junction restructuring, cytokines, proteases, and protease inhibitors^19^. Several other studies have reported alteration in spermatogenesis and consequent impairment of male fertility following aberration in the expression of genes required for these processes^15–18^.

Perusing the recent findings, it is clear that investigating the pathology, pathophysiology, and the general aetiology of male infertility should not only involve the basic analysis of semen parameters but should also include studying the association, interaction, and involvement of genes and protein expression in all stages of spermatogenesis, acrosome reaction, capacitation, and fertilization. Through reanalysis of the publically available transcriptomic database, the present study aimed to identify important and relevant genes that are essential for the process of spermatogenesis and overall male fertility. These genes may serve as potential biomarkers for early detection of NOA since NOA is mainly caused by impaired spermatogenesis, and the detection of these genes can furthermore aid treatment regimes. Additionally, the study strives to provide evidence that alteration in these genes' expression leads to male infertility.

## Methods

### Transcriptomics datasets selection

The gene expression omnibus (GEO) database was used (https://www.ncbi.nlm.nih.gov/geo/) to explore the common differentially expressed genes (DEGs) across different datasets of transcriptomic profiling of the testes from human patients with different causes of infertility/impaired spermatogenesis and/or azoospermia. For consistency and to decrease technical confounding factors, the following filters/criteria were used (i) datasets containing only human samples were selected, (ii) expression profiling by microarray for healthy controls and disease groups was used, and (iii) datasets having at least three samples per group were chosen. Among the 30 datasets available on infertility, only 5 fulfilled the inclusion criteria. The phenotype of the datasets as described by the studies include:

(i) GSE145467 (patients with impaired spermatogenesis (NOA; n = 10) and patients with normal spermatogenesis (OA; n = 10). This study regarded testicular samples collected from OA patients as control since they presented with complete spermatogenesis.
(ii) GSE108886 (patients with different types of azoospermia (NOA, n = 8; OA, n = 3; pooled control, n = 1).
(iii) GSE45887 (spermatogenic failure versus normal spermatogenesis. NOA patients are regarded as spermatogenic failure, n = 16; Controls are regarded as normal spermatogenesis, n = 4)
(iv) GSE45885 (NOA, n = 27; Control, n = 4).
(v) GSE25518 (testicular biopsy from Cryptorchid patients, n = 18; testicular samples (19 testicular biopsies from 18 boys) from contralateral descended testes, n = 4). Samples collected from patients with contralateral descended testes was considered as Control. This dataset was included because studies have shown that 32% of patients with bilateral and 10% with unilateral cryptorchidism will develop azoospermia. Cryptorchid boys at risk of azoospermia display a typical testicular histology of impaired mini-puberty at the time of the orchidopexy. To identify genes that may predict the future development of azoospermia in this disease, the authors (GSE25518) investigated the transcriptomic changes in the expression of genes in testicular samples from 19 cryptorchid boys and 4 testicular samples from patients with contralateral descended testes with normal spermatogenesis.
(vi) GSE9210 was the only dataset with no controls as it was designed to compare nonobstructive azoospermia (NOA; n = 47) to obstructive azoospermia (OA; n = 11) and was included as per Table 1.

**Table 1:** List of datasets that met the inclusion criteria.

Series number	Title	Samples	Organism
GSE145467	Transcriptome changes in patients with severely impaired spermatogenesis	Control (n = 10) Disease (Impaired spermatogenesis; n = 10)	Homo sapiens
GSE108886	Spermatogenomics: correlating testicular gene expression to human male infertility	Control (n = pooled) Disease (NOA, OA; n = 11)	Homo sapiens
GSE45887	The gene expression analysis of paracrine/autocrine factors in patients with spermatogenetic failure compared to normal spermatogenesis	Control (n = 4) Disease (NOA; n = 16)	Homo sapiens
GSE45885	Potential biomarkers of nonobstructive azoospermia identified in microarray gene expression analysis	Control (n = 4) Disease (NOA; n = 27)	Homo sapiens
GSE25518	Testis developmental gene expression in cryptorchid boys at risk of azoospermia	Control (n = 4) Disease (Cryptorchidism; n = 18)	Homo sapiens
GSE9210	A testicular gene expression profile for NOA patients, and ART3 as a genetic susceptibility gene for NOA	NOA (n = 47) OA (n = 11)	Homo sapiens

Experimental type = Expression profiling by array; NOA = non-obstructive azoospermia; OA = obstructive azoospermia.

### Identification of common DEGs

Identifying DEGs for each dataset was performed as previously described^20^. In brief, the GEOquery and limma R packages through the GEO2R tool for each dataset was used as follows^21^: The samples were grouped into diseases and healthy and then analyzed using the following parameters: applying log transformation to the data, applying limma precision weights (vooma), and force normalization. After sorting the genes according to the False Discovery Rate (FDR), the top differentially expressed probes with FDR < 0.05 were selected from each dataset. The annotated genes of the probes in each dataset were intersected with differentially expressed genes (DEGs) from all other datasets. The DEGs common in at least 4 out of 5 datasets (GSE145467, GSE108886, GSE45887, GSE45885, and GSE25518) were intersected with those from (GSE9210) and identified as shared genes that are consistent DEG in the testis of patients with infertility. Enriched Ontology Clustering for the identified genes was performed to explore the possible shared functional and mechanistic association and pathways using the Metascape (http://metascape.org/gp/index.html#/main/step1).

## Results

**Figure 1:**
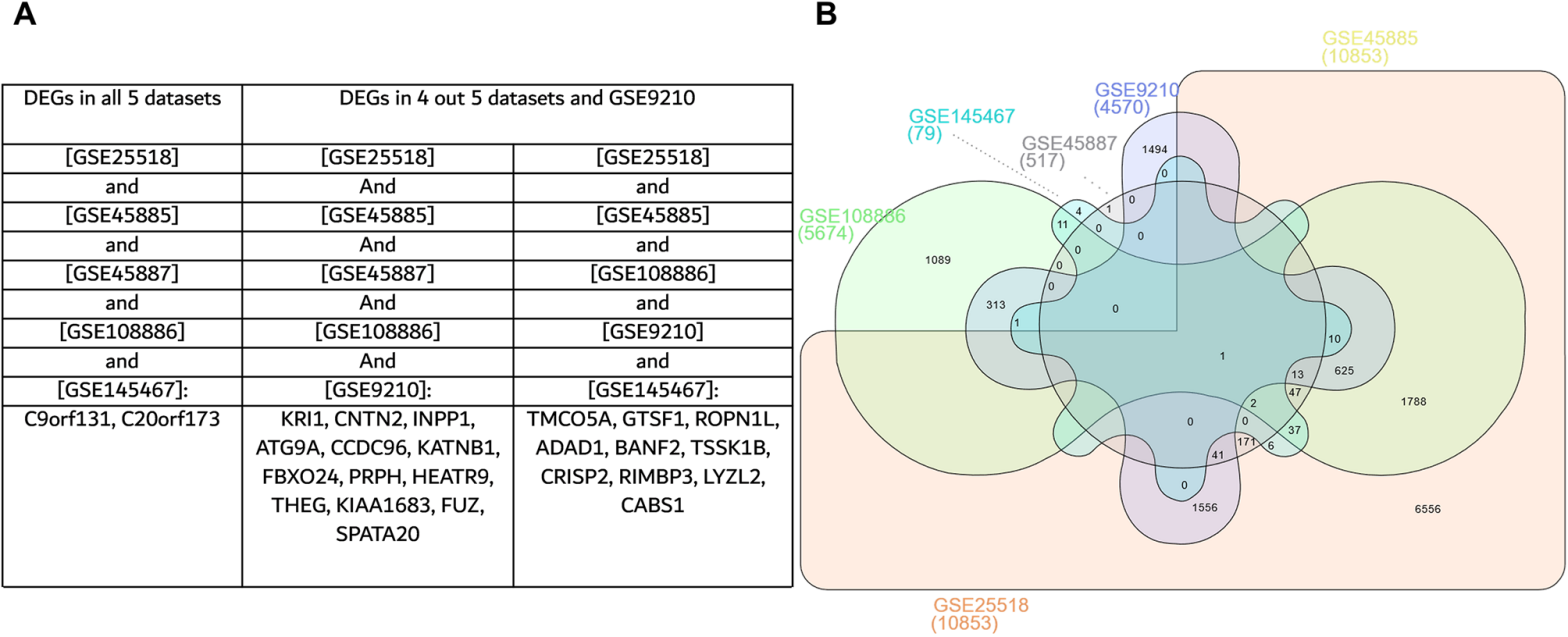
Identified differentially expressed genes in the testes shared between the datasets. (**A**) Tabular representation of the shared genes, (**B**) Venn diagram of the shared genes.

Our results showed that 25 DEGs were shared between most of the datasets (Fig. 1), which might indicate their role in the pathogenesis or are altered by the disease, making them interesting diagnostic biomarkers of the disease or players in its development.

GSE145467 investigated the transcriptomic changes in patients with impaired spermatogenesis (NOA) versus those with normal spermatogenesis (samples collected from OA patients). Twenty-three DEGs from the 25 were upregulated in patients with normal spermatogenesis when compared to the patients with impaired spermatogenesis. The LogFC of the DEGs are presented in Table 2. GSE108886 investigated the transcriptomic changes of gene expression in patients with different types of azoospermia. The study included 12 sample data, 1 pooled control and the remaining 11 include 8 samples from NOA patients and 3 from OA patients. Following analysis, No DEGs was observed between Control versus OA. The expression of these DEGs were significantly reduced in NOA patients compared to OA. The DEGs were significantly higher in the control group when compared to the NOA. This further shows that patients with OA may present with normal spermatogenesis, while patients with NOA usually show different abnormalities of spermatogenesis. Which can be pre-meiotic, meiotic, post-meiotic or Sertoli cell only syndrome. No DEGs was observed between Control, NOA and OA (Table 2). GSE45887 investigated transcriptomic changes in patients with normal spermatogenesis (control) and impaired spermatogenesis (patients with NOA of diverse etiologies). Study included 20 patients (control, n = 4; NOA, n = 16). The control group had significantly higher expression of these DEGs compared to the NOA group. Which means that the DEGs are downregulated in NOA (Table 2). GSE45885 investigated transcriptomic changes in patients with normal spermatogenesis (control) and impaired spermatogenesis (patients with NOA of diverse etiologies). (NOA = 27; Control = 4). These DEGs were significantly higher in control (normal spermatogenesis) group versus the NOA (Table 2). From the GSE25518 dataset, authors reported that a total of 483 genes were either under expressed or absent in the testes of cryptorchid boys that are likely to develop azoospermia, and these genes are involved in spermatogenesis. From our findings, 3 DEGs were highly expressed in the contralateral testes when compared to the cryptorchid group (Table 2). From dataset GSE9210, almost all the DEGs were downregulated in the NOA when compared to OA (Table 2).

**Table 2:** The Log fold change of the differentially expressed genes in all datasets.

List of DEGs	GSE145467	GSE108886		GSE45887	GSE45885	GSE25518	GSE9210
	NS versus IS	NOA versus OA	Control versus NOA	Control versus NOA	Control versus NOA	Contralateral testes (control) versus Cryptorchid	NOA versus OA
INPP1	2.139	− 1.686		1.218	1.293		− 1.068
ATG9A	1.011	− 0.734			1.32		0.706
CCDC96	2.071	− 1.841	1.675		1.197		− 1.51
THEG	0.991	− 2.343	1.86		2.387		− 0.189
SPATA20	1.654	− 1.173			1.828		
KRI1		0.336		0.872	0.865	0.907	− 0.569
CNTN2	0.85	− 0.897		1.471	1.569	0.431	− 0.462
KATNB1	1.468	− 1.185	1.258		1.448		− 0.172
FBXO24		− 1.123		2.095	2.11		− 2.067
PRPH	1.939	− 2.756	2.61		1.687		− 0.351
HEATR9	2.618	− 1.057		1.352	1.363		− 0.337
IQCN (KIAA1683)	1.891	− 1.545			1.614		− 0.578
C9orf131	4.415	− 1.436	1.438	2.589	2.658		
C20orf173	4.619	− 0.869		− 1.405	1.546		
TMCO5A	5.062	− 3.604	3.69			0.145	− 2.48
FUZ	2.392	− 1.000		0.67	0.707		
GTSF1	3.431	− 4.106	3.993		3.1		− 1.164
ROPN1L	3.59	− 5.077	4.704				− 2.408
ADAD1	4.917	− 3.638	3.914				− 2.461
BANF2	4.363	− 2.709	2.636				− 0.732
TSSK1B	4.512	− 2.297	2.043		1.554		− 1.478
CRISP2	4.922	− 5.788	5.861				− 2.631
RIMBP3	3.103	− 0.862			1.94		− 2.264
LYZL2	4.986	− 3.281					− 1.699
CABS1	4.662	− 3.544	3.592				− 0.769

DEGs = differentially expressed genes; NS = normal spermatogenesis; IS = impaired spermatogenesis; NOA = non-obstructive azoospermia; OA = obstructive azoospermia; Negative value (-) = downregulation, positive value = upregulation. Positive value GSE145467 = increased in normal versus impaired. The DEGs were significantly reduced in impaired spermatogenesis. From GSE108886, DEGs were significantly reduced in NOA when compared to OA and were also decreased when compared to control. That is, control samples show upregulation of the DEGs. DEGs were upregulation in control samples when compared to NOA in both GSE45887 and GSE45885, and they were downregulated when compared to OA (GSE9210). Some DEGs were upregulated in the contralateral testes when compared to the cryptorchid sample (GSE25518).

Taking a closer look at dataset GSE108886 (NOA vs OA) and dataset GSE9210 (NOA vs OA; a study that only investigated the testicular samples of azoospermic men), it is evident that these DEGs are downregulated in NOA, which further connotes the importance of the genes in the different phases of spermatogenesis. Having given a background on the general findings, the result for THEG is brought into focus to serve as an exemplar.

### THEG (THEG spermatid protein) is significantly downregulated in impaired spermatogenesis and infertility

The NOA group were divided into three histological subgroups, namely (i) NOA pre-meiotic arrest, (ii) NOA meiotic arrest, and (iii) NOA SCOS.

**Figure 2:**
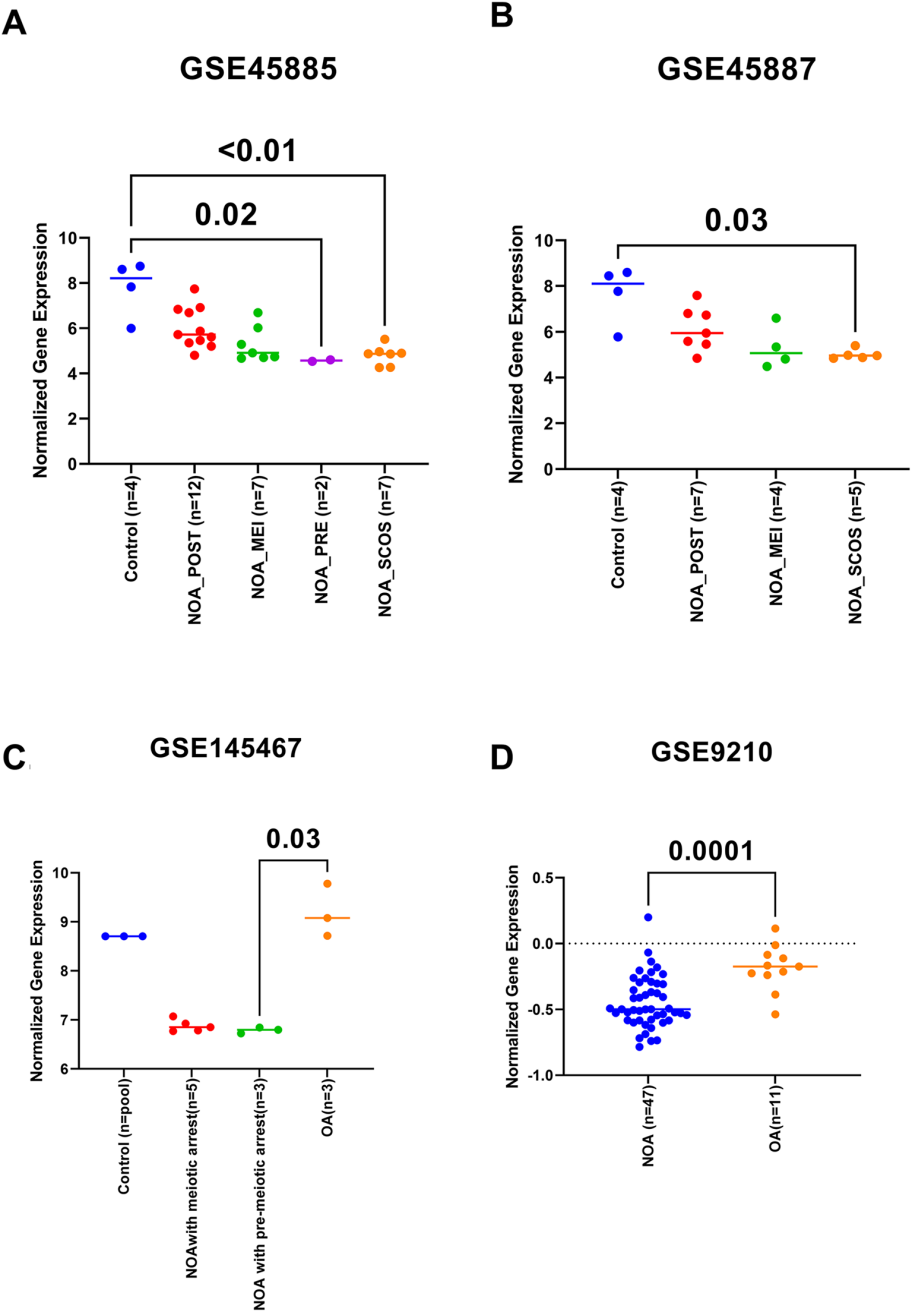
Findings for THEG. (**A**) expression of THEG from dataset GSE45885, (**B**) expression of THEG from dataset GSE45887, (**C**) expression of THEG from dataset145467, (**D**) expression of THEG from dataset 9210. The gene expression level of THEG was significantly reduced in patients with Sertoli-cell only syndrome (SCOS) in datasets GSE45885 and GSE45887 when compared to their respective controls. While the expression of THEG is significantly increased in OA group when compared to NOA (GSE9210). Since SCOS is one of the causes of NOA, then THEG expression was significantly reduced in NOA patients from GSE9210 dataset. NOA = non-obstructive azoospermia, OA = obstructive azoospermia, NOA-pre = NOA pre-meiotic arrest, NOA-mei = NOA meiotic arrest, NOA-post = NOA post-meiotic arrest.

To examine the dynamics of some of the identified genes in different types of samples and conditions taken in the datasets, the individual gene normalized gene expression in each dataset was extracted and plotted accordingly. In the first dataset (GSE45885 and GSE45887), THEG was downregulated in all disease samples of NOA compared to healthy. Its highest expression was in healthy controls followed by postmeiotic arrest (NOA_POST), meiotic arrest (NOA_MEI), premeiotic arrest (NOA_PRE), and Sertoli Cell Only-Syndrome (NOA_SCOS) (Fig. 2). Interestingly when comparing the THEG expression in (GSE145467), OA showed higher expression than the NOA samples. The same trend was documented in (GSE9210) which compared OA to NOA (Fig. 2).

### Discussion: The identified DEGs are involved in spermatogenesis and spermatid development

To understand the pathways where most of the 25 identified DEGs are involved, the genes were uploaded to the Metascape tool. It identified that most of the DEGs were involved in spermatogenesis (THEG, SPATA20, ROPN1L, TSSK1B, RIMBP3, CABS1, GTSF1, and ADAD1) and spermatid development (ROPN1L, TSSK1B, RIMBP3, and ADAD1) (Table 3).

**Table 3:** List of differentially expressed genes involved in spermatogenesis and spermiogenesis.


Category	Term	Description	LogP	Log(q-value)	Symbols
GO Biological Processes	GO:0007283	Spermatogenesis	− 7.73284	− 3.579	THEG, SPATA20, ROPN1L, TSSK1B, RIMBP3, CABS1, GTSF1, ADAD1
GO Biological Processes	GO:0007286	Spermatid development	− 4.92669	− 1.221	ROPN1L, TSSK1B, RIMBP3, ADAD1

Following gene enrichment ontology, eight genes (THEG, SPATA20, ROPN1L, TSSK1B, RIMBP3, CABS1, GTSF1, ADAD1) were considered to take part in the whole process of spermatogenesis, while four genes (ROPN1L, TSSK1B, RIMBP3, ADAD1) were shown to be involved in spermiogenesis.

**Figure 3:**
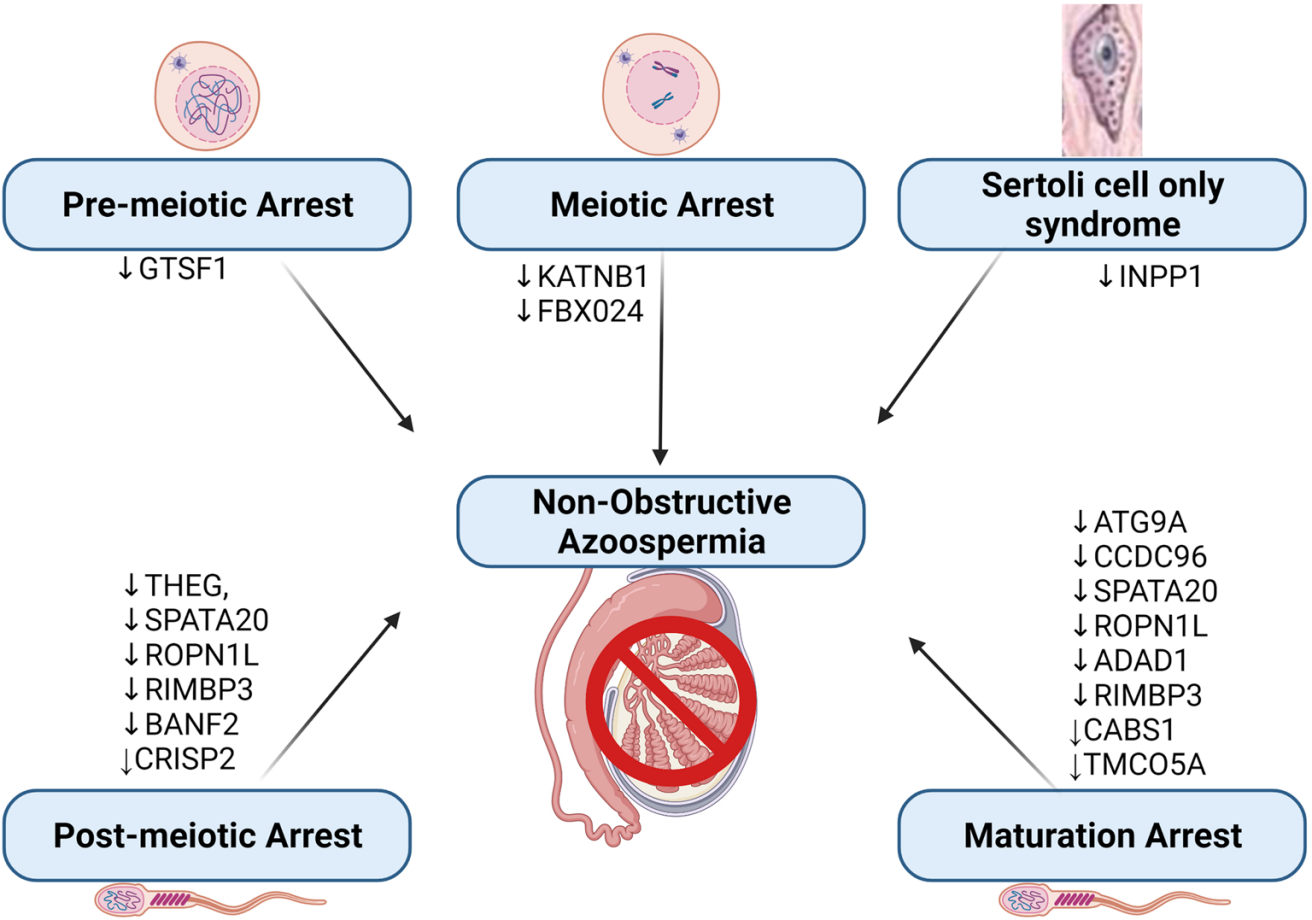
The role of some differentially expressed genes in the different histo-testicular abnormalities in non-obstructive azoospermia. NOA can occur as a result of pre-meiotic arrest, meiotic arrest, post-meiotic arrest or maturation arrest during spermatogenesis.

Subsequent to the detailed analysis of several datasets obtained from the GEO omnibus for possible diagnostic biomarkers involved in different phases of spermatogenesis, and in overall male infertility, the following genes/proteins were identified (INPP1, ATG9A, CCDC96, THEG, SPATA20, KRI1, CNTN2, KANTNB1, FBXO24, PRPPH, HEATR9, IQCN, FUZ, C9orf131, C20orf173, TMCO5A, GTSF1, ROPN1L, ADAD1, BANF2, TSSK1B, CRISP2, RIMBP3, LYZL2, CABS1). The importance of these genes will be briefly discussed, and their role in male infertility will be buttressed. The plausible stage-specific role of these DEGs is presented in Fig. 3.

### INPP1 (inositol polyphosphate 1 phosphatase)

The phosphatidylinositol signalling pathway is crucial in the regulation of different cellular processes, including cell metabolism, morphogenesis, cell cycle, cell polarity, cytoskeletal organization, and spermatogenesis^19,22^. The mechanistic regulation of these pathways involves controlled phosphorylation and de-phosphorylation of specific membrane-bound lipids and phosphatidylinositol phosphatases, situated at the 3–4-5 positions of the inositol ring^23^.

Inositol polyphosphate-1-phosphatase (INPP1) is an enzyme that cleaves 1-phosphate from inositol 1,4-biphosphate (Ins(1,4)P2) and inositol 1,3,4 triphosphate (Ins(1,3,4)P3. Although its impact as an antihypertrophic factor has been highlighted^24^, its role in spermatogenesis is yet understood. However, the role of inositol polyphosphate-4-phosphatase (INPP4) and inositol polyphosphate-5-phosphatase (INPP5) have been identified in spermatogenesis^22,25^. Ceyhan et al. reported that Inpp4b knockout mice presented with reduced testicular weight when compared to the wild-type and that these animals have lesser mature spermatozoa, which was further impacted with increased age^22^. Also reported were reduced steroidogenic enzymes, decreased expression of the LH receptor gene, and increased germ cell apoptosis. Therefore, it can be suggested that Inpp4b may play a role in the maintenance of germ cell differentiation. Another study reported the accumulation of abnormally swollen, actin-coated, endosome-like structures that contain intact adherens junctions and stains positive for N-cadherin and B-catenin in the Sertoli cells of mice deficient in Inpp5b^25^. Consequent to the formation of the abnormal Sertoli cell structure, developing spermatogenic cells were prematurely released from the seminiferous tubule and sloughed into the epididymis. These results collectively suggest the importance of phosphoinositides/polyphosphate phosphatases in spermatogenesis and that Inpp5b is important in the regulation of cell adhesion in the testes. Additionally, the abnormal formation of the Sertoli cells that occurs when this gene is absent, mutated or under expressed can be associated with the Sertoli cell-only syndrome that is seen in NOA.

### ATG9A (autophagy related 9A)

Autophagy is a lysosomal degradative process by which dysfunctional proteins and damaged organelles are degraded to maintain cell metabolism and energy homeostasis^26^. Autophagy is initiated with the formation of a phagophore, which then expands into a vesicle called an autophagosome. Autophagosome fuses with the lysosome to form an autolysosome. The process of autophagy is regulated by the autophagy-related genes (ATG)^27^.

Autophagy is involved in many physiological processes, including spermiogenesis and acrosome formation^26^. Studies have highlighted the importance of different ATGs in male fertility^28,29^. For instance, ATG5 and ATG7-mediated autophagy were shown to play a role in differentiation and cytoplasmic reduction of the flagellated motile sperm^29^, while Huang et al. presented that ATG5 is required for elongating spermatid development, sperm individualization, and normal fertility in male mice^26^. Another study showed that germ cell-specific disruption of ATG7 causes altered acrosome formation and abnormal sperm morphology in mice^30^. Asgari et al. reported the decreased expression of ATG5 in the semen sample of azoospermic patients. The importance of ATG5 has been described in the Sertoli and Leydig cells of the testes^31,32^. Although the precise role of ATG9A in male fertility is yet to be elucidated, a study reported that the loss of ATG9A function triggers ovarian failure (primary ovarian insufficiency) in COS-7 cells^27^. Another study reported that the overexpression of AT-1(a systemic membrane transporter) led to reduced fertility and that this may be due to the blockage of ATG9A^33^. Furthermore, the results of the current study showed that ATG9A expression was downregulated in the testicular samples collected from NOA men, depicting that it may play a role in spermatogenesis, especially during spermiogenesis.

### CCDC96 (coiled-coil domain containing 96)

The centriole is a microtubule-based structure that is preserved in all eukaryotes that makes cilia^34^. Sperm centrioles are essential for two main functions, including (i) the formation of the sperm flagellum required for movement and (ii) the development of the embryo after fertilization. The sperm supplies the centriole that creates the centrosome and microtubule system of the zygote. Hence, centriole abnormalities may be a cause of male factor infertility^35^. Firat-Karala et al. reported the presence of some genes/proteins that are important for centriole function^34^. Assessment of bovine sperm sample revealed centrosome-associated proteins, which include CCDC96 and others(CCDC113, C4orf47, CCDC38, C7orf31, CCDC146, CCDC86, and CCDC116). From the current study, the transcriptomic expression of CCDC96 was downregulated in the testicular samples of NOA men. CCDC96 has been previously described to play a role in ciliary beating and the deletion thereof alters beating frequency^36^. Shamseldin et al. reported that alteration in CCDC96 and other centriole-associated proteins led to ciliopathies^37^. Since sperm has two distinctive centrioles involved in motility, modification in the centriole-linked proteins such as altered CCDC96 may result in altered sperm motility and, subsequently male infertility.

### THEG (testicular haploid expressed gene)

Spermatogenesis is a developmental process, which occurs within the seminiferous tubule of the testes and is regulated by an enormous number of proteins. In the seminiferous tubule of the testis, spermatogenic cells are in constant communication with the Sertoli cells, and this interaction is essential for germ cell differentiation^38^. The co-culture experiments have enabled the identification of a wide array of proteins secreted by the Sertoli cell that influences germ cell differentiation. One of the identified proteins is the testicular haploid expressed gene (THEG). THEG is known to be expressed in spermatids, and the availability of this protein is regulated by the Sertoli cell^38^. The co-culture of Sertoli cells and spermatids revealed the expression of THEG at a basal level; however, when spermatids were cultured separately, the expression of THEG was reduced, suggesting that the Sertoli cell is required for spermatid THEG homeostasis. This is supported by an animal study conducted by Nayernia et al., with their findings showing the relevance of germ cell-Sertoli cell interaction for gene regulation during spermatogenesis^38^.

Although the exact role of THEG in spermatid is not fully understood, it was reported that the mutants of THEG in drosophila were sterile^39^. At the same time, another study showed that transgenic mice with disrupted THEG had abnormal germ cell development with the presence of abnormally elongated spermatids in the lumen of the seminiferous tubule. Contrary to the above studies, Mannan et al. reported that mice lacking THEG had normal spermatogenesis, except that the animals presented with reduced testicular size^40^. At this point, we can say that findings on the exact role of THEG in male fertility are controversial, but the hypogonadism observed cannot be over sighted. Additionally, the fact that it is reduced in NOA as shown from our findings further buttress the point that its role in spermatogenesis cannot be overemphasized.

### SPATA20 (spermatogenesis associated 20)

Spermatogenesis-associated gene family (SPATA) are thioredoxin-like proteins, and they play an important role in spermatogenesis, sperm maturation, and fertilization^41^. They also play a part in acrosome formation^42^, maintain mitochondrial function^43^, sperm production, and sperm motility^44^. In order to study the pathway through which SPATA20 specifically affects male fertility, Zheng et al. investigated its role in sperm DNA hydroxymethylation of men exposed to bisphenol A (BPA)^15^. An increase in sperm 5-hydroxymethylcytosine levels (5hmc, a product in the intermediate process of DNA demethylation) was observed, with elevated genome regions harbouring 5hmc, and elevated maternally expressed imprinted genes in BPA-exposed sperm. BPA exposure negatively affected gene expression in sperm, including SPATA20. Hence, it is suggested that SPATA20 may participate in response to DNA damage in sperm, thereby affecting sperm quality. Another study implicated SPATA20 as one of the testicular genes that were altered in obese mice^45^. The consequence of these modified proteins was a reduction in the number of Sertoli cells and an alteration in the structure of spermatids, which occurs partly due to upregulation in testicular oxidative stress. Sujit et al., on the other hand, reported hypermethylation of SPATA4, SPATA5, and SPATA6 in the semen sample of oligozoospermic patients^41^. It can be suggested that hypermethylation of these genes affected some pathways involved in sperm production. From the current study as well, SPATA20 was downregulated in NOA and was upregulated in OA compared to NOA. This further buttress the importance normal SPATA20 expression in spermatogenesis since some OA patients may present with normal spermatogenesis^46^. These findings taken together, it is clear that alteration in the function of SPATA20 may result in reduced sperm quality and ultimately male infertility.

### KATNB1 (Katanin regulatory subunit B1)

Katanin is a preserved microtubule-severing complex consisting of two units. The first, p60, is an enzymatic subunit encoded by the katanin regulatory subunit A1 (katna1) gene, while the second, p80, is an accessory protein subunit encoded by the katanin regulatory subunit B1 (katnb1) gene. The accessory protein subunit regulates the microtubule-severing complex^47–49^. Katanin exerts its effect by binding to a microtubule to form a complex. This complex undergoes a conformational change upon ATP hydrolysis, de-stabilizing the tubulin-tubulin contacts within the microtubule lattice, leading to microtubule severing^47–49^. Katanin plays a role in cell division and the development of neurons^50^. Interestingly, both subunits of katanin (p60-katna1 and p80-katnb1) were shown to play a role in the fertility capacity of male mice^51^.

A study reported that mice that are katnb1 mutant displayed reduced epididymal sperm count, and there were defects in the meiotic division, which lead to a decrease in the number of spermatids and defects in spermiation. The animals also presented with low sperm motility and increased abnormal sperm morphology, characterized by head deformities, which was due to defect in the development and function of the manchette^52^. The manchette is a structure required for sperm head shaping, and alteration in its function results in abnormal sperm morphology relating to head defects^53,54^. Additionally, both katna1 and katnab1 were present on microtubule-based structures within developing germ cells, including the meiotic spindle, the axoneme, and the manchette. However, these microtubule-based structures were abnormal in mutant mice, indicating that katnb1 mutation may impair male fertility. To further validate animal studies, genomic DNA samples of both fertile and infertile men (oligoasthenoteratozoospermic) were analyzed/screened for katnb1 genetic variations. Direct DNA genome sequencing reveals the presence of 10 katnb1 variants in the semen samples of OAT men, which was absent in the control group^52^. Due to the lack of statistical significance in the expression of these ten variants, it was suggested that genetic variants in the katnb1 gene are not commonly associated with men presenting with oligoasthenoteratozospermia within their study population.

Pleuger et al. reported katanin 80 (katnb1) expression in both normal and impaired seminiferous tubules of human testicular biopsy samples. Katnb1 mRNA was expressed in germ cells, spermatogonia entering meiosis, and in the Golgi complex of pachytene spermatocytes^55^. Looking at the known role of katanin (both katna1 and katnb1) during spermiogenesis and spermiation, a thorough investigation should be performed to elucidate further the impact of katnb1 alteration in spermatogenesis and the overall impact/effect on male fertility. Understanding its role in the meiotic phase of spermatogenesis is especially important in patients with meiotic-arrest azoospermia.

### FBXO24 (F-box protein 24)

F-box proteins function as substrate adaptors for the S-phase kinase-associated protein 1 (SKP1)- cullin 1 (CULL1)-F-box protein (SCF) ubiquitin ligase complexes. They control the proteasomal degradation of a wide array of regulatory proteins^56^.

F-box proteins consist of two main functional domains: (i) F-box motif and (ii) a carboxyl-terminal domain. F-box motif is a protein–protein interaction domain that recruits F-box proteins to the SKP1-CULL1-F-box protein (SCF) E3 ligase complex through direct binding to the adaptor protein SKP1^57^, while the carboxy-terminal domain binds to specific substrates.

F-box proteins have several subunits, including FBXO1, FBXO24, FBXO43, FBXO47, FBXW7. It is important to note that studies on F-box proteins relating to male infertility are limited. Hence, a brief overview of the available data on the different subunits of F-box proteins will be highlighted.

Rong et al. showed that FBXO47 regulates the telomere-shelterin complex that is specifically expressed during meiotic prophase 1. As FBXO47 knockout mice presented with deficient spermatocytes with spermatocytes unable to form a complete synaptonemal complex. It was also reported that during normal spermatogenesis FBX047 interacts with TRF1/2, however, after the knockout of FBXO47, there was a disruption of this interaction, which de-stabilizes TRF2 and subsequently lead to unstable telomere attachment and slow transversing through the bouquet stage^58^. Taken together, fbxo47 is important for the process of spermatogenesis.

The main role of FBXO43 is to act as a cytostatic factor, a component that is required for the maintenance of metaphase II arrest in mice oocytes^59^. It is also important for the process spermiogenesis; as spermatocytes did not undergo complete meiotic division, and as well essential for spermatogenesis, as there was an absence of spermatids in the testes of FBXO43 knockout mice^60^. Ma et al. reported the mutation of FBXO43 in the DNA sample of teratozoospermic infertile men, and the mutations were absent in fertile controls^61^. It was concluded that the mutation of FBXO43 is a causative factor of male infertility and teratozoospermia. Deducing from the trend of expression of the other subunits of FBX proteins in altered spermatogenesis, the down regulation of FBX024 in the NOA group of the current study indicate that this gene may play a vital role during the meiotic phase of spermatogenesis.

### HEATR9 (HEAT repeat containing 9)

HEAT repeat containing 9 (HEATR9) is an infection-responsive gene that affects cytokine production in alveolar epithelial cells^62^. During infection, viruses enter into susceptible host cells to replicate their component to produce new virions. In the infection process, the gene expression of infected cells changes because of the formation of the virions. A recent study confirmed the in vitro and in vivo expression of HEATR9 during viral infection^62^. It was reported that the knockdown of HEATR9 during infection affected chemokine expression. Hence, HEATR9 is said to be involved in inflammatory and virus infections serving as a regulator of specific cytokines. HEAT repeats are protein structural motifs of approximately 37–43 residues in length, and they occur in protein sequences with anywhere from 3 to more than 20 repeats. HEAT is derived from four proteins that contain multiple HEAT repeats, including (i) huntingtin, (ii) elongation factor 3, (iii) protein phosphatase 2A, and (iv) mammalian target of rapamycin 1. There are no available studies on its impact on male infertility; however, studies investigating the impact or effect of testicular infection or inflammation such as orchitis may explore the role of HEATR9 and how this can help with future therapeutics.

### IQCN (IQ motif containing N)

IQ motifs are present in various proteins, including myosins and a variety of non-myosin proteins, such as phosphatases, voltage-gated channels, neuronal growth proteins, spindle-associated proteins, and sperm surface proteins^63^. IQ motifs-containing proteins are characteristically associated with calmodulin (CaM) regulation. There are ten known IQ-motifs-containing flagellar proteins^64^. CaM is a calcium sensor and functions as a regulator while interacting with diverse cellular proteins. Although there are no available studies on the association between IQCN and male infertility, a few studies have elucidated the involvement of IQCG in spermatogenesis; spermiogenesis specifically^65,66^.

IQCG is one of the diverse IQ motive containing genes. In the oligoasthenoteratozoospermia (OAT) mouse model of male infertility, Harris et al*.* reported that the loss of IQCG disrupted spermiogenesis such that tail formation either occurs incompletely or breaks apart from the sperm head^65^. Another study also showed that the expression of IQCG spans through the human testes, from the spermatogonia (strongly expressed) through to the spermatocytes and spermatids (specifically in the tail region)^67^. It was suggested that IQCG might play a role in human sperm motility, and its reduced expression may impair sperm motility and thus affect overall male fertility. These results collectively showed that IQ-motifs containing proteins are crucial for spermatogenesis and the maintenance of male fertility.

### FUZ (fuzzy planar cell polarity protein)

The fuzzy planar cell polarity protein (FUZ) is an effector component of the planar cell polarity (PCP) signalling. PCP pathway is a conserved signalling axis that organizes the polarized movement of cells within a planar plane to achieve patterning and morphogenesis^68^. Studies have elucidated the relationship between PCP signalling and mammalian embryonic neural development, as the genetic alteration of PCP genes leads to fatal mal-neurodevelopmental issues^69,70^. FUZ knockout mice displayed severe developmental retardation accompanied by ciliogenesis impairment and malfunctioning of several important developmental pathways^71^. FUZ was also reported to have a pro-apoptotic function, as the overexpression of FUZ stimulated the DVL-Racl-MAPK-Caspase signalling pathway to trigger cell apoptosis^72^. Thus, its role in cancer has also been reported^73^; however, its role in spermatogenesis or infertility is yet to be shown.

### GTSF1 (gametocyte specific factor 1)

Gametocyte-specific factor 1 (GTSF1) belongs to a functionally uncharacterized protein family (UPO224) with an unknown functional domain^74^. This family consists of three members, including GTSF1(also expressed in humans), GTSF1L, and GTSF2, which are expressed in germline cells in mice. These genes generate proteins with two repeats of the CHHC zn-finger domain, a predicted RNA-binding motif in the N terminus^75^. GTSF1 is localized on chromosome 15 in mice and on chromosome 12q13.2 in humans. In some species, GTSF1 is required for the silencing of retrotransposons through the Piwi (P-element-induced wimpy testes) interacting RNA (piRNA) pathway^76^.

Spermatogonia contain processing bodies that harbor Piwi proteins. Piwi proteins are associated mainly with Piwi-interacting RNAs to silence transposable DNA elements. Loss of function/mutations in the Piwi pathway lead to derepression of transposable elements, resulting in altered spermatogenesis^16^ Deletion of GTSF1 causes male-specific sterility and derepression of LINE-1 retrotransposons^77^. A decrease in the GTSF1 expression was observed in testicular biopsy of boys with cryptorchidism^16^.

A study assessed the presence of GTSF1 gene in human oocytes and preimplantation embryos. GTSF1 was detected in the ovarian follicles, germinal vesicle stage oocytes, metaphase II oocytes, the morula, and blastocyst stage preimplantation embryos. GTSF1 was also observed in fetal ovaries during gestation from 8 to 21 weeks, while GTSF1 was expressed in human fetal testis from 8 to 19 weeks of gestation^76^. The presence of these genes even in fetal gonads suggests its conservation may be important for fertility. Another study measured the expression of GTSF1 during embryonic development and its localization during gonadal development and gametocyte maturation in mice. GTSF1 mRNA expression was observed in both male and female gonads, localizing to germ-cells throughout development. The expression of GTSF1 during gonadal development suggests it may play an essential role in germ cell development^74^.

Yoshimura et al. also reported that GSF1 protein is localized in spermatozoa. In order to assess the specific role of gtsf1 in spermatogenesis, they measured the GTSF1 expression in the testes of GTSF1-knockout mice. It was reported that although these mice grew normally and appeared healthy, they were sterile due to massive apoptotic death of the germ cells after postnatal day 14. It was further revealed that GTSf1 knockout mice had ceased meiotic division progression before the zygotene stage^77^. Additionally, the expression of GTSF1 in the NOA group of the current study was downregulated, showing the plausible role during proliferation and differentiation. These results suggest that lack or reduced expression of GTSF1 is may negatively impact spermatogenesis and over all male fertility.

### ROPN1L (Rhophilin associated tail protein 1 like)

In mammals, rhophilin associated tail protein 1 like (ROPN1L) and the orthologous ROPN1 are located in the fibrous sheath of sperm. The fibrous sheath is a flagellar cytoskeletal structure unique to sperm, and it surrounds the dense outer fibers and the axoneme. The main component of the fibrous sheath is the A-kinase anchor proteins (AKAPs; AKAP3 and AKAP4). Both ROPN1Land ROPN1binds AKAP3 via an N-terminal located domain present in other proteins^78,79^. Although the biochemical actions of ROPN1L are mainly unknown, it was suggested to regulate the activity of AKAP3, such as cAMP-dependent protein kinase^80^. Defects in sperm morphology were observed in the testes of ropn1l and ropn1 knockout mice, with a reduction in AKAP3 levels^81^. Ropn1l knockout mice displayed mildly impaired sperm motility, while ROPN1L and Ropn1 knockout mice had immotile spermatozoa.

Additionally, spermatozoa retrieved from ropn1l knockout mice showed a reduction in cAMP-dependent protein kinase phosphorylation. This is supported by the study of Zhang et al., with the inclusion that ROPN1L and ROPN1may be involved in sperm capacitation^82^. Another study also reported the presence of ROPN1L on the spermatozoa tail of *Opisthorchis viverrini* and that the sera of individuals suffering from opisthorchiasis showed reactivity to this protein^80^. The presence of ROPN1L on the spermatozoa tail suggests it may play a role in sperm motility.

These results collectively suggest that ROPN1L mutation may result in defects in fibrous sheath integrity, reduced sperm motility, altered protein kinase signalling pathway, which may ultimately lead to male infertility.

### ADAD1 (Adenosine deaminase domain containing 1)

RNA-editing is also known as the irreversible chemical modification of a nucleotide within an intact RNA^83^. RNA-editing is a group of post-transcriptional modifications that aids the complexity of the transcriptome^84^. There are two main types of RNA-editing; (i) Adenosine to Inosine and (ii) cytosine to uridine; of which adenosine to inosine occurs more frequently^85^. Adenosine-to-inosine RNA editing is a fundamental RNA modification regulated by adenosine deaminase (AD) domain-containing proteins.

The AD proteins in humans include AD RNA-specific 1 and 2 (ADAR1 and ADAR2). These enzymes contain one double-stranded RNA binding motif and an AD domain, which catalyzes the conversion of adenosine to inosine^86^.

In the process of identifying other RNA editing regulatory pathways, tissue-specific AD domain proteins were revealed. This includes ADAD1 and the other nine proteins. ADAD1 is testes specific and has been shown to be essential/required for normal male fertility^87^. ADAD1 is localized in round spermatids, while ADAD2 is predominantly expressed in mid-to-late pachytene spermatocytes, indicating they may play a role in meiotic and postmeiotic germ cell RNA editing^83^. The role of ADAD1 in spermatogenesis has also been shown by several other authors^88,89^.

Mutation of either ADAD1 or ADAD2 resulted in males sterility with ADAD1 mutant mice displaying teratozoospermia. This further suggests that alteration in the expression of ADAD1 may impair spermatogenesis and male fertility at large.

### TSSK1B (testis specific serine kinase 1 B)

The testis-specific serine kinases (TSSKs) are formed within the calcium/calmodulin-dependent protein kinase superfamily^90^. In mammals, more than five TSSKs have been identified^91,92^. In the mice model, Tssk1–4 and Tssk6 were specifically expressed in the testis at the postmeiotic phase^92^. The deletion of both Tssk1 andTssk2 resulted in male chimaeras carrying the mutant allele in spermatogenic cells, but this allele was not transmitted to the offspring, indicating infertility because of haploinsufficiency^93^. Another study reported that Tssk1/2 knockout mice have a testis-specific characteristic, with late spermatids showing developmental dysregulation of the formation of the mitochondrial sheath, which subsequently resulted in male infertility^94^.

In humans, TSSK1A gene is located in tandem with TSSK2 within the DiGeorge Syndrome region on chromosome22q11.21^95^. Another testicular kinase identified in humans is the TSSK1B that is located on human chromosome 5q22.2^91^. It has been shown that TSSK1B may interact with TSSK2 to obtain a sufficient dose of TSSK1B/TSSK2 total kinase activity in developing spermatids. TSSK1B has been shown to play a role in the different phases of spermatogenesis and sperm maturation, including cytodifferentiation of spermatids, spermiation, and acquirement of sperm fertilizing capacity^91,94^. Cai et al. also showed that TSSK1B is essential for proper spermatogenesis in cattleyak, most especially at the meiotic phase^96^.

Deletion of tssk1b gene was detected in a patient with asthenoteratozoospermia, and further analysis of the AZF region on the Y-chromosome showed no microdeletion, suggesting that tssk1b is important for male fertility^97^. These findings indicate that TSSK1B is important for spermatogonial development, cell differentiation, spermiogenesis, and spermiation. This means that alteration or mutation of this gene may lead to male fertility impairment.

### RIMBP3 (Rims binding protein 3)

Rim-binding proteins (RIM-BPs) were identified to have a structural and functional link between the presynaptic active zone proteins RIMs, and as well for the voltage-gated ca^2+^ channels^98^. RIMs form an integral part of the cytomatrix at the presynaptic active zone and are coupled to synaptic vesicles through their interaction with the small GTPase Rab3^99^. RIM-BPs are large multidomain proteins, and they constitute a novel class of proteins. All RIM-BPs contain three SH3-domains, two to three contiguous fibronectin type III domains, and isoform-specific regions at the N-terminus and between the clustered domains. RIM-BP3 is exclusively expressed in mammals and is encoded by a single exon, of which three copies are present in the human genome^100^.

Jing et al. reported the specialized function of rimbp3 during spermiogenesis in mice. RIM-BP3 protein is associated with the manchette, a transient microtubular structure considered to be important for morphogenesis during spermiogenesis. RIM-BP3 knock-out mice presented with abnormal sperm heads, characterized by a deformed nucleus and a detached acrosome. It was added that rimbp3 is associated with Hook1, a known manchette-bound protein required for sperm head morphogenesis and that no association was detected when Hook1 protein was truncated/modified (Hook1 knock out mice). When both rimbp3 and hook1 were deleted in mice, an ectopic positioning of the manchette within the spermatid was observed, a presumed cause of sperm head deformities^101^. The findings indicate the essential role of rimbp3 in sperm head formation through its interaction with hook1, and an alteration in the expression of this gene may result in male fertility impairment.

### CABS1 (calcium binding protein, spermatid associated 1)

The calcium-binding protein spermatid-associated 1 (Cabs1) is a calcium-binding protein specifically expressed in elongated spermatids of mice^102^, human^103^, porcine^104^, and rat testes^105^. The Cabs1 protein is localized in the acrosome and principal piece of the flagellum of mature sperm in the cauda epididymis. It was suggested that porcine Cabs1 might be involved in the acrosome reaction by controlling calcium signalling^103^. Cabs1 can be phosphorylated by a serine-threonine kinase (Casein kinase 2) that caused sperm deformities^105^. During the process of identifying proteins that are specifically expressed at different stages of spermatogenesis, Akihiro et al. for the first time reported the expression of CABS1 in elongated spermatids of mice testes and its detection in the principal piece of mature sperm flagellum in the caudal epididymis^102^.

Xiaoning et al. reported that the Cabs1 knock-out mice did not display testicular and epididymal development impairment; however, the sperm tail structure was significantly impaired, and subfertility ensue. Further analysis revealed defects in sperm flagellar differentiation, which lead to an abnormal annulus and disorganization of the midpiece–principal piece junction, and may in part explain the high percentage of sperm with a bent tail. Intriguingly, the proportion of sperm with a bent tail increased during transit in the epididymis^106^. These findings indicate that Cabs1 forms an important part of the sperm annulus which is essential for proper sperm tail assembly and motility. This further suggests that deletion or mutation of the gene may result in male infertility.

### C9orf131 (chromosome 9 open reading frame 131) and C20orf173 (chromosome 20 open reading frame 173)

Although studies on the association between C9orf131, C20orf173, and male fertility have not been established, few studies have revealed the mutation of C9orf72 in patients with dementia^107^, and amyotrophic lateral sclerosis^108^. Studies investigating the association of these genes with azoospermia, oligozoospermia, asthenozoospermia, and ultimately male infertility should be developed, as this may further help in unraveling idiopathic infertility.

#### TMCO5A (transmembrane and coiled coil domains 5A)

Kaneko et al. reported the expression of transmembrane and coiled-coil domains 5A (TMCO5A) in rat testes from the 4th week of postnatal development. The distribution of TMCO5A was further shown in the endoplasmic reticulum-nuclear membrane (ER-NM) of cells, acting as a membrane-associated protein, while TMCO5A cells lacking a transmembrane region were mislocalized and were diffused through the cytoplasm. This suggests that the transmembrane region is responsible for the retention of TMCO5A at the ER-NM^109^. TMCO5A was also found in the posterior part of nuclei in both round and elongated rat spermatids and is closely associated with developing manchette microtubules. This collectively suggests that TMCO5A may be important for the formation of the sperm head and hence is essential for sperm motility^109^.

### BANF2 (barrier-to-autointegration factor-like protein 2)

Barrier-to-autointegration factor-like protein 2 (BANF2), also called BAFL, is a nuclear protein that is expressed in spermatids and are retained in mature ejaculated spermatozoa^110^. BANF2 is densely expressed in the testes, and it is hypothesized to control BANF1 function, which plays a role in chromatin structure, segregation, and post-mitotic nuclear assembly^111^. Pini et al. reported that BANF2 is involved in spermatogenesis and mature sperm function following a proteomic analysis of the sperm retrieved from obese men. The authors reported a significant reduction in the spermatozoa expression of BANF2 in obese men and these men were asthenozoospermic and had altered acrosome reaction^112^. Other studies have reported reduced zona pellucida binding^113^ and a slower pronuclear fusion to achieve syngamy following fertilization^114^. Another study reported the detection of BAFL (BANF2) and BAF expression in ejaculated spermatozoa^115^. Taken together, this indicates that BANF2 may play an essential role in the maintenance of sperm nuclear shape as well as in the formation of the male pro-nucleus following fertilization.

### CRISP2 (cysteine rich secretory protein 2)

CRISP2 is a member of the cysteine-rich secretory protein (CRISP) family of the cysteine-rich secretory protein/antigen 5/pathogenesis (CAP) superfamily^116^. It is highly expressed within the testis, and it is specifically localized to the sperm acrosome, accessory structures of the sperm tail, and the junction between germ and Sertoli cells within the seminiferous epithelium^117^. Similar to all CRISPs, CRISP2 consists of a CAP domain, which is implicated in cell–cell adhesion and is capable of steroid binding, and a CRISP domain. The CRISP domain can be further subdivided into (i) a hinge region (which is required to maintain spatial separation between the CAP and CRISP domains), and (ii) an ion channel regulatory region (which in the case of CRISP2 can specifically regulate calcium flow through ryanodine receptors)^118^.

A study assessed the expression of CRISP2 in the spermatozoa and seminal plasma fluid of three groups of infertile men (asthenozoospermia, teratozoospermia, and teratoasthenozoospermia), and then compared with the control (normozoospermic infertile men). They reported that CRISP2 expression was significantly reduced in the spermatozoa and seminal plasma of all three groups^119^. This, therefore, suggest that there is an association between the level of CRISP2 expression and male infertility. Another study assessed the expression of CRISP2 in the sperm of idiopathic asthenozoospermic men, a significant reduction in the expression of CRISP2 in the spermatozoa of these men was reported, indicating that this protein may be involved in the regulation of sperm functional parameters, especially, sperm motility^120^. This was supported by other studies^121,122^. Jun-Hao et al. revealed that the reduced CRISP2 expression in the spermatozoa of asthenozoospermic patients is regulated at a post-transcriptional level. As microRNA-27b (a regulator of CRISP2) was highly expressed in the ejaculated spermatozoa of these men and was negatively correlated with the expression of CRISP2. These findings suggest that the reduced CRISP2 seen in asthenozoospermia is probably caused by an alteration in the regulation of CRISP2 regulator^122^.

Lim et al. assessed the role of CRISP2 in male fertility in a mouse model. They reported that CRISP2 aid spermatozoa to undergo acrosome reaction and also help in forming a normal flagellum waveform. As CRISP2 knockout mice presented with a stiff midpiece and are thus unable to show the rapid form of progressive motility seen in the control^118^. The data collectively suggest that CRISP2 is essential for sperm motility, and its deficiency may lead to male subfertility. Several other animal studies have revealed the role of CRISP2 in spermatogenesis, motility, acrosome reaction, and fertilization^123,124^, with the inclusion that CRISP2 interact with sperm head and tail associated protein (SHTAP) during mouse spermatogenesis^125^ and with gametogenetin1 for sperm tail development^126^.

### LYZL2 (lysozyme like 2)

Testis specific lysozyme-like genes (Lyzl) belong to the c-type lysozyme family. Five substrates of lysozyme-like genes have been identified in the testis of human and other mammalian species, this includes Lyzl2, Lyzl3/Spaca3, Lyzl4, Lyzl5/Spaca5, and Lyzl6^127–129^. The LYZL have also been identified from sperm proteome of mouse and human^130,131^. LYZL4 protein has been identified on the surface of human embryonic stem cells^132^. LYZL4, LYZL3, and LYZL5 have also been shown to be expressed in the human sperm tail proteome^133^. Additionally, Zhang et al. reported the expression of four lysozyme-like genes in the testis and epididymis^127^. Further analysis of LYZL4 gene revealed that its mRNA was only found in the epithelium of human epididymides, most abundantly in the caput, suggesting that LYZL4 plays a physiological role in male reproduction.

### KRI1 (KRI1 homolog)

KRI1 is an ortholog of KRIT1/CCM1, which is mutated in human neurovascular disease cerebral cavernous malformation (CCM)^134^. KRI1 is required for DNA damage-dependent cell death independently of Cep-1/p53^135^. There are no available data on the role of KRI1 in male fertility; however, KRI1 has been shown to integrate signals from the germ cells of *C. elegans* to elicit longevity effects in non-reproductive tissues^136^. Studies addressing the role of KRI1 in male reproduction should be developed, seeing that its mutation leads to CCM.

### CNTN2 (Contactin 2)

Contactin (CNTN) belongs to the immunoglobulin superfamily and comprises of six members (CNTN1-6).CNTN2 is also referred to as Tag1, and it is a neural-specific glycoprotein, which functions as a cell adhesion molecule anchored within the cell membrane^18^. CNTN2 is a synaptic and axonal membrane protein that interacts with proteins involved in the pathology of Alzheimer's disease^137^. Chatterjee et al. reported reduced CNTN2 in the cerebrospinal fluid of AD patients, and this positively correlated with BACE1 (a protein involved in AD). The findings suggest that decreased CNTN2 cerebrospinal fluid (CSF) levels may be a biomarker for synaptic or axonal loss in AD^137^. Although studies on CNTN2 and male fertility are currently not available, the role of contactin-associated protein-like 2 (CACNA2D1) has been briefly identified^138^. Li et al. reported a DNA fragment that breaks from chromosome 7 and inserts into chromosome 18 with inversion in an azoospermic patient. Although this patient presented with a normal hormonal profile and no microdeletion of the Y chromosome, the semen sample was still void of sperm. It was reported that the azoospermia was associated with insertional translocation with karyotype of 46XY, inv ins (18,7) (q22.1;q36.2q21.11) and that at the chromosomal breakpoints, CACNA2D1 and DPP6 were disrupted^138^. Taken together, it can be suggested that the aberration or disruption of CACNA2D1 can be a biomarker for azoospermia.

## Summary

The use of conventional semen analysis for the diagnosis and treatment of male infertility has become insufficient; hence there is a need to widen the scope of investigating the aetiology of male infertility. The current study used publicly available transcriptomic data to identify DEGs that are of importance in NOA and overall male fertility. Twenty-five DEGs were identified across five different datasets and are shown to be involved in impaired spermatogenesis and male infertility. Following further analysis, eight of these DEGs are implicated in NOA, serving as potential biomarkers of the disease as they specifically play vital roles in spermatocytogenesis, spermiogenesis, spermiation, and morphogenesis. Some of which are also important in sperm motility, capacitation, and gonadal development, hence, are essential for male fertility.

**Figure 4:**
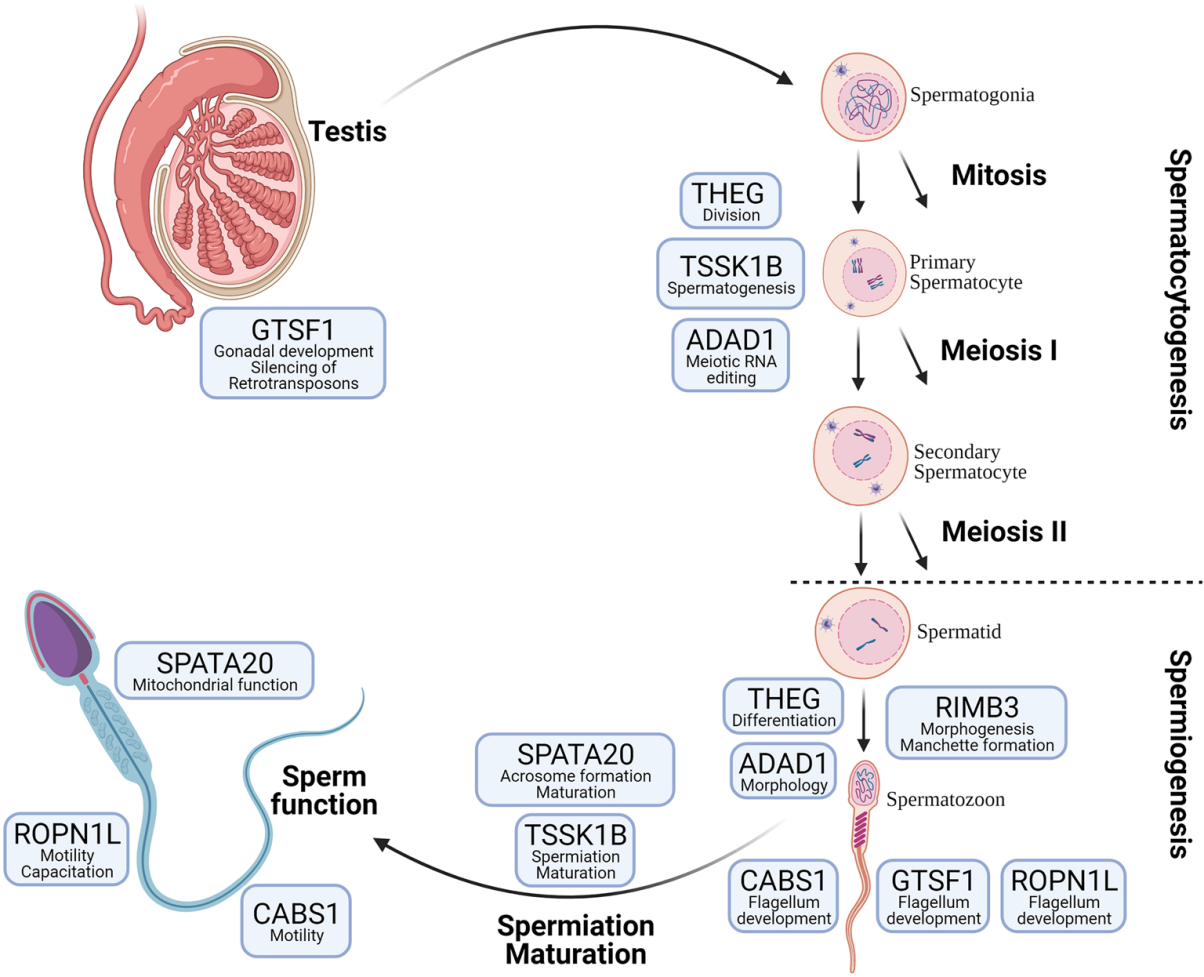
Summary of the functions of the selected differentially expressed genes. Briefly, THEG, TSSK1B, and ADA1 are involved in spermatocytogenesis, with ADAD1 playing a role in the meiotic and postmeiotic germ cell RNA editing. RIMBP3, CABS1, GTSF1, ADAD1, and THEG are involved in developing different spermatozoa structures. RIMBP3 is involved in manchette formation, for normal sperm morphology, CABS1, ROPN1L, and GTSF1 are involved in flagellum development and normal sperm motility. SPATA20 is involved in acrosome formation and maintains mitochondria function during spermatogenesis, while SPATA20 and TSSK1B are both critical for sperm maturation.

Represented in Fig. 4 is the summary of the functions and the involvement of these selected genes (THEG, TSSK1B, ADAD1, RIMBP3, CABS1, GTSF1, ROPN1L, SPATA20). In brief, THEG, TSSK1B, and ADA1 are involved in spermatocytogenesis, with ADAD1 playing a role in the meiotic and postmeiotic germ cell RNA editing. RIMBP3, CABS1, GTSF1, ADAD1 and THEG, are involved in the development of different spermatozoa structures. RIMBP3 is involved in manchette formation; for normal sperm morphology, CABS1, ROPN1L and GTSF1 are involved in flagellum development; for normal sperm motility, SPATA20 is involved in acrosome formation, maintains mitochondria function during spermatogenesis, while SPATA and TSSK1B are both important for sperm maturation.

The importance of analysing transcriptomic data to understand the role of genes in various pathways, and the further application of the results can be seen in a study conducted by Fu et al. (2019). The group described the glycolysis metabolic changes in sperm cryopreservation based on a targeted metabolomic strategy. The potential metabolomic pathway changes in the sperm cryopreservation process and new markers for human sperm freezability were reported. After identifying 16 compounds which were significantly deregulated between fresh and post-thawed sperm (7 were downregulated and 9 were upregulated in the frozen thawed group), a bioinformatics analysis was performed. Analysis revealed that metabolic pathways play an important role in cryopreservation. The pathways include the citrate cycle (TCA cycle), glycolysis or gluconeogenesis, glyoxylate and dicarboxylate metabolism, pyruvate metabolism, to mention a few. Authors then performed wet experiments to elucidate the importance of substrates specific to the identified pathways, as revealed by the Insilco analysis. Results from protein quantification confirmed the previous genomic analysis of the metabolites, and the results confirmed the differential protein levels observed^139^. Thus, validating the importance and the translatability of Insilco data.

Conclusively, the normal expression and interaction of these genes are required for normal spermatogenic processes and ultimately for male fertility. It is, therefore, recommended that future studies investigating NOA and the pathophysiology of male infertility, particularly idiopathic male infertility, should consider the impact of altered gene expression with an initial focus on the genes identified/highlighted in this study.
